# Emergence of a New Highly Successful Acapsular Group A *Streptococcus* Clade of Genotype *emm*89 in the United Kingdom

**DOI:** 10.1128/mBio.00622-15

**Published:** 2015-07-14

**Authors:** Claire E. Turner, James Abbott, Theresa Lamagni, Matthew T. G. Holden, Sophia David, Michael D. Jones, Laurence Game, Androulla Efstratiou, Shiranee Sriskandan

**Affiliations:** ^a^ Infectious Diseases and Immunity, Department of Medicine, Imperial College London, London, United Kingdom; ^b^ Bioinformatics Support Service, Department of Life Sciences, Imperial College London, London, United Kingdom; ^c^ Public Health England, London, United Kingdom; ^d^ Pathogen Genomics, The Wellcome Trust Sanger Institute, Cambridge, United Kingdom; ^e^ School of Medicine, University of St. Andrews, St. Andrews, United Kingdom; ^f^ MRC Clinical Sciences Centre, Hammersmith Hospital, London, United Kingdom; Northern Arizona University

## Abstract

Group A *Streptococcus* (GAS) genotype *emm*89 is increasingly recognized as a leading cause of disease worldwide, yet factors that underlie the success of this *emm* type are unknown. Surveillance identified a sustained nationwide increase in *emm*89 invasive GAS disease in the United Kingdom, prompting longitudinal investigation of this genotype. Whole-genome sequencing revealed a recent dramatic shift in the *emm*89 population with the emergence of a new clade that increased to dominance over previous *emm*89 variants. Temporal analysis indicated that the clade arose in the early 1990s but abruptly increased in prevalence in 2008, coinciding with an increased incidence of *emm*89 infections. Although standard variable typing regions (*emm* subtype, *tee* type, *sof* type, and multilocus sequence typing [MLST]) remained unchanged, uniquely the emergent clade had undergone six distinct regions of homologous recombination across the genome compared to the rest of the sequenced *emm*89 population. Two of these regions affected known virulence factors, the hyaluronic acid capsule and the toxins NADase and streptolysin O. Unexpectedly, and in contrast to the rest of the sequenced *emm*89 population, the emergent clade-associated strains were genetically acapsular, rendering them unable to produce the hyaluronic acid capsule. The emergent clade-associated strains had also acquired an NADase/streptolysin O locus nearly identical to that found in *emm*12 and modern *emm*1 strains but different from the rest of the sequenced *emm*89 population. The emergent clade-associated strains had enhanced expression of NADase and streptolysin O. The genome remodeling in the new clade variant and the resultant altered phenotype appear to have conferred a selective advantage over other *emm*89 variants and may explain the changes observed in *emm*89 GAS epidemiology.

## IMPORTANCE

Sudden upsurges or epidemic waves are common features of group A streptococcal disease. Although the mechanisms behind such changes are largely unknown, they are often associated with an expansion of a single genotype within the population. Using whole-genome sequencing, we investigated a nationwide increase in invasive disease caused by the genotype *emm*89 in the United Kingdom. We identified a new clade variant that had recently emerged in the *emm*89 population after having undergone several core genomic recombination-related changes, two of which affected known virulence factors. An unusual finding of the new variant was the loss of the hyaluronic acid capsule, previously thought to be essential for causing invasive disease. A further genomic adaptation in the NADase/streptolysin O locus resulted in enhanced production of these toxins. Recombination-related genome remodeling is clearly an important mechanism in group A *Streptococcus* that can give rise to more successful and potentially more pathogenic variants.

## INTRODUCTION

The human pathogen *Streptococcus pyogenes* or group A *Streptococcus* (GAS) accounts for over 600 million infections globally per year with a high level of morbidity and mortality (1). Frequently observed upsurges in GAS disease are associated with the emergence and expansion of a new *emm* genotype or the sudden increase of a preexisting common *emm* type. The mechanisms behind such epidemic waves of disease are largely unknown but may in part be due to the transference of new virulence factors between strains, mediated by mobile genetic elements such as bacteriophages commonly found in the GAS population.

Recent epidemiological evidence points to a rapid emergence of the GAS genotype *emm*89 as a leading cause of disease in the United Kingdom and other parts of the world, particularly Canada (2). Indeed, globally, *emm*89 is now among the top five leading *emm* types, equally capable of causing both invasive and noninvasive disease (2–13), as well as outbreaks (14–16). We identified a sustained rise in invasive GAS (iGAS) disease caused by *emm*89 strains in the United Kingdom over the past decade. Indeed, *emm*89 strains have remained among the top three *emm* types causing iGAS and occasionally have even overtaken the consistently dominant *emm*1. Despite the global prevalence of *emm*89, it is a relatively understudied *emm* type. Here, we report the first genomic study of *emm*89 GAS and reveal the emergence of a new *emm*89 clade variant that had undergone homologous recombination of core genomic regions. The new emergent clade variant was unexpectedly genetically acapsular and exhibited enhanced production of the toxins NADase and streptolysin O (SLO). Furthermore, the emergent acapsular clade variant increased in the population, temporally associated with the rise in *emm*89 iGAS, and is now the dominant *emm*89 variant in the United Kingdom population.

## RESULTS

### An increase in *emm*89 iGAS.

**FIG 1:**
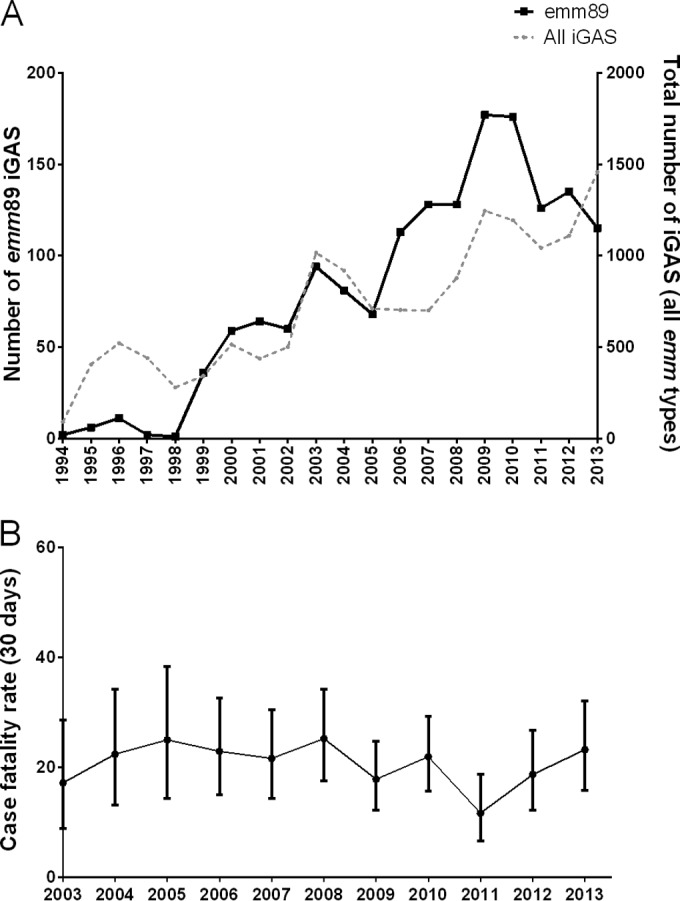
United Kingdom epidemiology of *emm*89 invasive GAS disease (iGAS). (A) Incidence of *emm*89 iGAS (black solid line, left axis) alongside the total numbers of iGAS cases of all *emm* types (gray dotted line, right axis). (B) Thirty-day case fatality rates of *emm*89 iGAS (±95% confidence interval).

National surveillance of iGAS in England and Wales, supported by serological and then molecular typing of iGAS-associated strains, identified a sustained increase in disease caused by strains of the *emm*89 lineage from 1998 to 2013. While the rise in *emm*89 strains between 1999 and 2005 was broadly in proportion with the overall rise in iGAS infection, a disproportionate rise was evident from 2005 to 2009; *emm*89 case numbers increased 2.6-fold compared to 1.8-fold for all other *emm* types combined (Fig. 1A). The proportion of all iGAS infections due to *emm*89 increased from 10% in 2005 to 18% in 2007. Between 2003 and 2013, 7-day and 30-day case fatality rates attributable to *emm*89 iGAS disease were 14% and 21%, respectively; while year-to-year fluctuation in case fatality rates was observed, there were no significant temporal trends (Fig. 1B).

### A high level of single nucleotide polymorphisms (SNPs) defines a novel clade of *emm*89 GAS.

The sustained increase in prevalence of *emm*89 iGAS coupled with the lack of *emm*89*-*specific genomic information regarding *emm*89 virulence provided the rationale for whole-genome sequencing (WGS) of 131 *emm*89 clinical GAS strains. Sequenced strains included 9 to 11 *emm*89 isolates from each year 2004 to 2013 comprising similar numbers of invasive (58/131) and noninvasive (73/131) isolates, randomly selected from those submitted to the national reference laboratory from different locations throughout the United Kingdom (see Table S1 in the supplemental material). To provide a reference *emm*89 genome for comparative analysis, one clinical necrotizing fasciitis isolate, H293, was sequenced to completion and annotated (GenBank accession no. HG316453.2).

**FIG 2:**
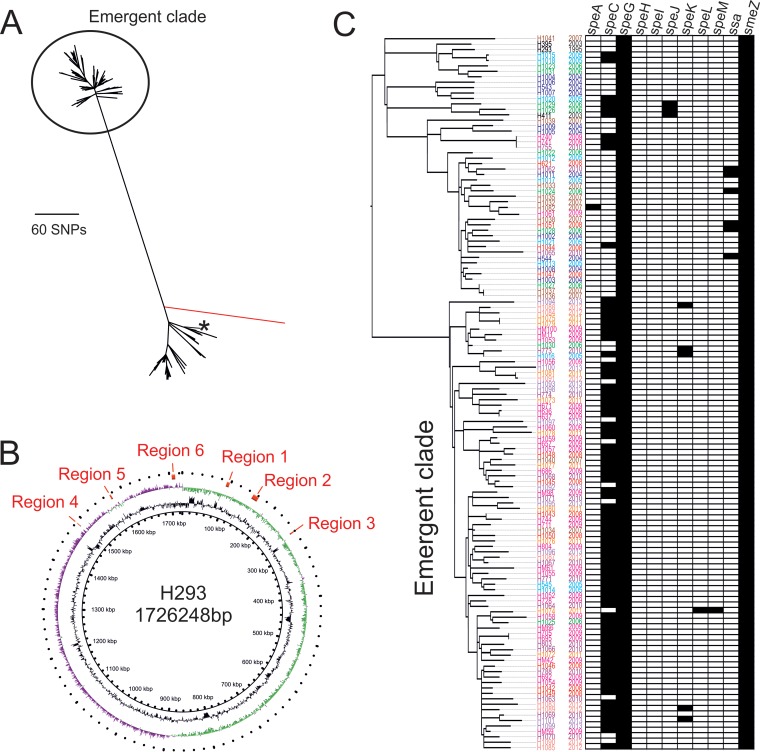
Phylogenetic analysis of all 131 *emm*89 isolates sequenced. (A) Maximum likelihood unrooted tree based on core SNPs compared to the reference strain H293. Eighty-three of 131 isolates clustered into a distinct emergent clade (circled) separate from the 47/131 isolates that clustered with the reference strain H293 (indicated by asterisk). A potential hypermutator strain, H1041, is indicated by a red branch. (B) Schematic of the H293 genome. The outer ring indicates the positions of the six regions of recombination present in emergent clade-associated *emm*89 strains relative to H293 and all other non-clade-associated *emm*89 strains (regions 1 to 6 shown in red). The inner two rings represent GC skew and GC content. (C) Mid-rooted phylogenetic tree based on core genome SNPs compared to the reference strain H293 excluding all regions of predicted recombination. The emergent *emm*89 clade is distinct from the rest of the diverse *emm*89 population. Strains are color coded by year of isolation. The presence of each of the 11 different superantigen genes is indicated to the right of the tree.

Mapping of the short read sequences generated from WGS for all 130 additional *emm*89 isolates to the completed H293 reference genome identified 2,075 single nucleotide polymorphisms (SNPs). Surprisingly, an SNP-based phylogenetic reconstruction revealed a separate clade of isolates within the *emm*89 population, distinct from isolates that clustered close to the reference strain H293 (Fig. 2A). Invasive and noninvasive GAS infection isolates were equally represented across the phylogeny. The clade was characterized by 229 SNPs, shared by all members of the clade compared to all other sequenced *emm*89 strains. Within the clade, there were additional polymorphisms; pairwise analysis of clade-associated strains showed that they differed by an average of 80 SNPs, while, outside the clade, strains differed by an average of 57 SNPs. Strain H1041 appeared to be a potential hypermutator strain with 223 SNPs compared to the reference strain, 180 of which were unique to this strain (Fig. 2A).

Sequence typing information was extracted from *de novo* assemblies of the WGS isolates but identified no clade-associated differences; all 131 strains were *emm* subtype *emm*89.0, multilocus sequence type 101, serum opacity factor type *sof89*, and T-antigen gene type *tee11*. Analysis for SNP clustering and sites of potential recombination revealed that the genomic distribution of the 229 SNPs characterizing the clade was not even (see Fig. S1 in the supplemental material). The majority of SNPs (202, 88%) clustered into six distinct regions (regions 1 to 6), located around the chromosomal origin of replication (Fig. 2B). These regions were within the core genome and unassociated with any potential mobile genetic elements. Within regions 1 to 6, the ratio of nonsynonymous to synonymous SNPs (dN/dS) was ~0.3, yet outside these regions the dN/dS ratio was ~1.8. The higher proportion of synonymous to nonsynonymous mutations within the six regions is consistent with recombination and indicates diverged donor lineages (17).

### Evolution of *emm*89 GAS in United Kingdom population.

**FIG 3:**
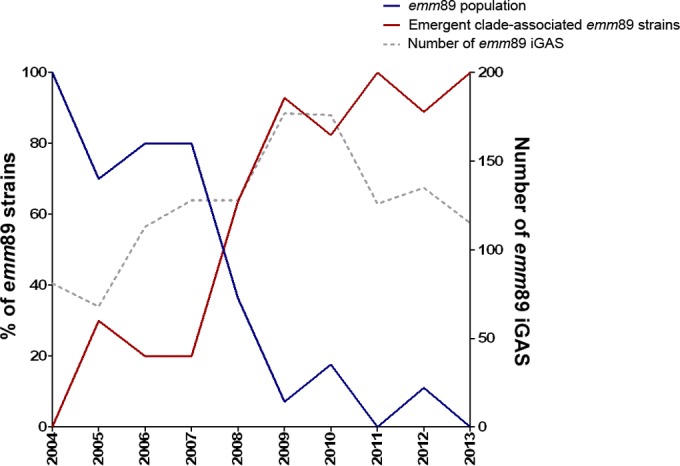
Rise of the emergent clade to dominance in the United Kingdom *emm*89 population. Between 2004 and 2007, the United Kingdom was represented by a diverse *emm*89 population (blue line, left axis); however, there was a sudden switch between 2007 and 2008 and the emergent clade-associated *emm*89 variant (red line, left axis) became the dominating type in the population. The rise of the emergent clade coincided with an increase in the frequency of *emm*89 iGAS in the United Kingdom (gray dotted line, right axis). Association with the emergent clade was determined by WGS of 126 *emm*89 strains (excluding pairs of strains isolated from the same patient) isolated in 2004 to 2013 (*n* = 10 for 2005, 2006, 2007, 2011, and 2013; *n* = 11 for 2004 and 2008; *n* = 28 for 2009; *n* = 17 for 2010; *n* = 9 for 2012).

Temporal investigation of the *emm*89 population structure by genomic and phylogenetic analysis revealed a dramatic national shift. The core SNP phylogeny, excluding all regions of recombination, indicated that the United Kingdom had hosted a diverse *emm*89 population, from which a distinct clade emerged (Fig. 2C). By 2008, the emergent clade had increased in the population and became dominant over any previous United Kingdom *emm*89 variant (Fig. 3). To investigate the population dynamics of *emm*89 strains, we performed temporal Bayesian analyses (BEAST) using SNPs identified in the core genome, excluding regions of predicted recombination. This revealed that the time to most recent common ancestor (tMRCA) of the whole *emm*89 population was approximately 1970 (2 May 1970; 95% highest probability density [HPD], 22 December 1962 to 7 July 1977) (see Fig. S2A in the supplemental material). The estimated substitution rate for the population was 7.99 × 10^−7^ site^−1^ year^−1^ (95% HPD, 6.58 × 10^−7^ to 9.35 to 10^−7^ site^−1^ year^−1^), corresponding to an evolutionary rate of 2.1 SNPs per genome per year, similar to rates determined for other GAS *emm* types (18, 19). Linear regression of maximum likelihood root-to-tip distances against the year of sampling showed a strong correlation with these data (see Fig. S2B). Using the Bayesian phylogenetic reconstruction, we were able to estimate that the tMRCA of the emergent clade was approximately 1992 (22 June 1992; 95% HPD, 13 August 1988 to 10 June 1996). Based on the phylogenetic framework and the temporal calibration, it would appear that the six recombination regions which are uniquely present in the emergent clade were acquired at some point in the ~20-year period prior to its emergence, i.e., between the tMRCA of the emergent clade and the last ancestral node shared with the rest of the population (7 September 1973; 95% HPD, 30 May 1967 to 28 July 1980). We predict that the recombination occurred in a step-by-step process due to the relatively dispersed location of the regions on the chromosome; however, we have not been able to identify any intermediate strains that have fewer than the six regions of recombination present, possibly because they have been lost in the population. Since the emergence of the new clade in the early 1990s, the population has expanded to become the dominant *emm*89 subclone.

### Prophage-like element with superantigen gene *speC* associated with majority of emergent clade-associated strains.

**FIG 4:**
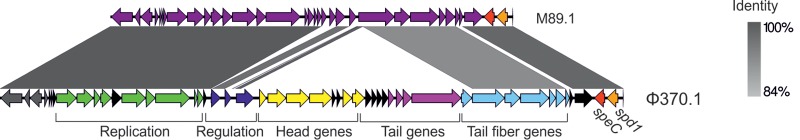
Prophage-like element found in the emergent clade-associated strains. In clade-associated strains, the superantigen gene *speC* (shown in red) and the DNase gene *spd1* (shown in orange) were associated with a prophage-like element (ΦM89.1) that shared a high level of identity to a prophage found in the genome of M1 strain SF370 (Φ370.1). Bacteriophage structural genes found in Φ370.1 (20) are absent in ΦM89.1. The figure was drawn using Easyfig (52).

The genomic complement of up to 11 known streptococcal superantigen genes can be variable as, with the exception of *speJ*, *speG*, and *smeZ*, superantigen genes are associated with potentially mobile bacteriophages. Notably, however, 71/83 strains of the emergent clade carried *speC* (86%) compared to only 10/48 strains outside this clade (21%) (Fig. 2C). All 71/83 *speC*-positive clade-associated strains carried the same prophage-like element with *speC* and a DNase gene, *spd1*, commonly found associated with *speC*. This prophage-like element was not found in any strains outside the clade; strains outside the clade with *speC* carried the toxin gene on a different prophage, of which there have been several identified for GAS. The emergent clade-associated *speC*-*spd1* prophage-like element (named ΦM89.1) was similar to that found in M1 GAS strain SF370 (Φ370.1) (Fig. 4); however, the predicted phage structural genes from Φ370.1 (20) were absent in ΦM89.1, suggesting that ΦM89.1 cannot form a lysogenic phage particle. While ΦM89.1 was common among the emergent clade-associated strains, the phage was not universally present and did not characterize the clade.

### Phenotypic impact of recombination-related remodeling.

We hypothesized that recombination-related genome remodeling led to the emergence of the new *emm*89 clade variant and provided a selective advantage over previous *emm*89 variants through altered phenotype and/or enhanced pathogenesis. The SNPs present in regions 1, 3, 4, and 5 (Table 1) were unlikely to result in any phenotypic changes as the few, if any, nonsynonymous SNPs present in these regions were predicted not to affect protein structure or function. Two regions, however, affected known GAS virulence factors: the NADase/streptolysin O toxin locus (region 2) and the hyaluronic acid (HA) capsule locus (region 6).

**TABLE 1:** Genes located within recombination regions 1 to 6 and associated single nucleotide polymorphisms^a^

Region	Gene	Function	No. of mutations			AA change(s)
			S	NS	Total/total in region	
1	*pbpb1b*	Penicillin binding protein	7	2	9/23	Ile430Val, Val537Ile
	*rpoB*	DNA-directed RNA polymerase	14	0	14/23	
2	00183	Hypotheticalprotein	1	2	3/130	Arg3Iso,Ala67Val
	*purA*	Adenylosuccinate synthetase	45	5	50/130	Gly84Ala, Thr213Ala, Leu348Phe, Lys351Glu, Tyr355His
	00185	Nucleoside-binding protein	5	1	6/130	Ala332Thr
	*nusG*	Transcription antitermination protein	3	0	3/130	
	*nga*	NAD glycohydrolase	11	6	17/130	Ala99Val, His103Arg, Arg136Gly, His143Gln, Met221Iso, Gln253His
	*ifs*	Immunity factor for SPN/NGA	2	2	4/130	Gly7Ser, Ala136Val
	*slo*	Streptolysin O	15	8	23/130	Thr39Ala, Iso59Thr, Ala130Thr, Met172Arg, Asp182Asn, Asp324Glu, Thr450Ser, Arg470Gln
	*metB*	Cystathionine beta-lyase	2	1	3/130	Val241Ala
3	00285	d-Alanyl-d-alanine carboxypeptidase	6	2	8/9	Iso27Met, Tyr364Gln
	*dacA.1*	d-Alanyl-d-alanine carboxypeptidase	1	0	1/9	
4	*lacR.2*	Lactose phosphotransferase system repressor	9	0	9/16	
5	01551	ABC transporter ATP-binding protein	5	0	5/9	
	01552	ABC transporter	2	2	4/9	Asp269Gly, Asp408Gly
6	01673	Zn-dependent peptidase	1	1	2/15	Iso169Val
	01677	Hypothetical protein	1	3	4/15	Thr67Iso, Iso83Met, Gln115Lys
	*recF*	DNA replication and repair protein	4	4	8/15	Ala59Glu, Gln246His, Asn263Asp, Val363Iso

^a^ Abbreviations: S, synonymous; NS, nonsynonymous; AA, amino acid (position in relation to reference H293 strain).

### Genomic region 2: effects on NADase locus/streptolysin O.

**FIG 5:**
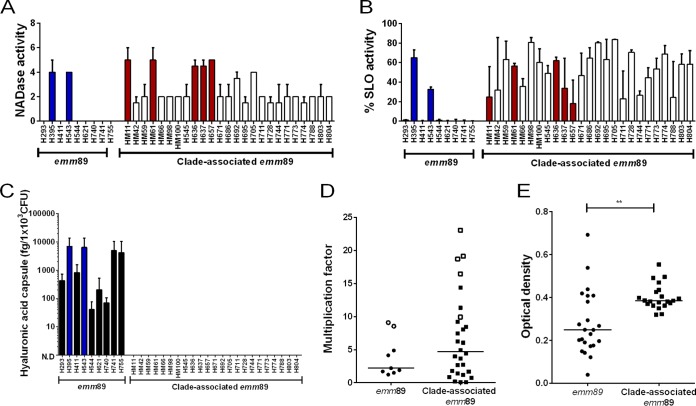
Variation in phenotype between *emm*89 strains. (A) NADase activities of 34 sequenced *emm*89 strains were measured in the culture supernatant; the result shown is the highest 2-fold dilution able to hydrolyze NAD^+^. Data represent the median (plus range) from four independent experiments. (B) SLO activities of 34 sequenced *emm*89 strains were measured in the culture supernatant by lysis of sheep erythrocytes and reported as the percentage of activity relative to the positive control (H_2_O). Data represent the means (+ standard deviations) from three independent experiments. (C) Production of hyaluronic acid was measured using an ELISA-based assay for hyaluronan. Representatives of the non-clade-associated *emm*89 strains tested all had detectable levels of hyaluronic acid. All representatives of emergent clade-associated strains tested had undetectable levels of hyaluronic acid (N.D., not detected). Data represent the means (+ standard deviations) from three independent experiments. Hyaluronic acid capsule was measured as femtograms per 1 × 10^3^ CFU. (D) Both non-clade-associated and clade-associated *emm*89 strains survive equally well in whole human blood. There was no overall difference in multiplication factor between the two types of strains, including (as shown) or excluding (not shown) strains with *covR/S* or *rocA* mutations. Strains were grown in duplicate using a single donor, and data represent the means. The experiment was repeated in a second donor with similar results. (E) The ability of invasive non-clade-associated strains (*n* = 23) and emergent clade-associated strains (*n* = 21) to adhere to and colonize uncoated plastic after 24 h of culture was measured by staining with Gram’s crystal violet. Data represent the means from three experiments. **, *P* ≤ 0.01, Mann-Whitney comparison. Strains with blue bars or unfilled circles (non-clade-associated *emm*89 strains) and red bars or unfilled squares (emergent clade-associated *emm*89 strains) carry mutations in *covR/S* or *rocA* known to control expression of *nga*, *slo*, and hyaluronic acid capsule; mutations in either *covR/S* or *rocA* would enhance *nga*, *slo*, and capsule expression.

The gene *nga* encodes NADase, a secreted toxin that cleaves β-NAD^+^, an essential component of many energy-producing reactions. NADase enters host cells through pores made by the coexpressed streptolysin O, encoded by *slo*; thus, they work in combination to produce a toxic effect on host cells (21, 22). Compared to all non-clade-associated *emm*89 strains, the emergent clade-associated strains contained 17 SNPs within *nga*, six of which were nonsynonymous, and 23 SNPs in *slo*, eight of which were nonsynonymous. All sequenced *emm*89 strains were predicted to have a functional NADase and SLO despite the SNPs within coding regions (23–25). Remarkably, although all clade-associated strains tested were capable of hydrolyzing NAD^+^, only two non-clade-associated strains tested had detectable NADase activity (Fig. 5A). A Western blotting assay probing for NADase indicated that this difference in activity was accounted for by enhanced protein expression of NADase by clade-associated strains (see Fig. S3 in the supplemental material).

Similar to NADase, all emergent clade-associated strains tested demonstrated high activity of SLO (Fig. 5B) in contrast to non-clade-associated strains tested, only two of which had SLO activity. Some emergent clade-associated strains tested (HM11, HM61, H636, H637, and H657) demonstrated enhanced NADase and SLO activity that could possibly be related to mutations in the regulatory kinase gene, *covS*. The two-component system CovR/S is known to negatively regulate the expression of the *nga/ifs/slo* locus, which in turn is modulated by the regulator of *cov*, *rocA*, which influences *covR/S* regulation (26, 27). Interestingly, the SLO and NADase activity of the two unusual non-clade-associated strains could also be potentially due to a mutation in *covS* and a deletion of the *rocA* gene in strains H395 and H543, respectively.

The virulence of globally dominant modern *emm*1 strains has been attributed to the acquisition of a 36-kb genomic region possibly from *emm*12 strains that includes the *nga*-*slo* locus and led to subsequent enhancement of SLO and NADase expression (19, 25). A comparison of the *emm*89 *nga*-*slo* locus (and surrounding 12-kb sequence SPYH293_0083 to *metB*) with all sequenced GAS genomes available demonstrated that the sequence in clade-associated *emm*89 strains shares 99% DNA identity with that of modern *emm*/M1 strains and *emm*/M12 strains (see Fig. S4 in the supplemental material). Whether this reflects recombination between *emm*89 and *emm*1 or *emm*89 and *emm*12 GAS is unclear, but recombination has resulted in a potentially advantageous increase in NADase/SLO production by the emergent clade-associated strains that recapitulates the production observed with modern *emm*/M1 strains (19, 25).

### Genomic region 6: effects on the synthesis of the hyaluronic acid capsule.

Region 6 contained five genes, three of which are required for synthesis of the hyaluronic acid (HA) capsule: *hasA*, *hasB*, and *hasC*. In the emergent clade-associated strains, the entire *hasABC* locus, including the promoter region, was completely absent and in place of this locus was a short region of 157 bp in length. The 157-bp sequence was not found anywhere else in the *emm*89 genome or in any other sequenced GAS genomes except for MGAS10750, an M4 strain that also lacks the *hasABC* locus (28). Interestingly, the same 157-bp sequence is also present in the completed genomes of *Streptococcus dysgalactiae* subsp. *equisimilis* in what appears to be a homologous region (see Fig. S5 in the supplemental material).

HA production was measured using an enzyme-linked immunosorbent assay (ELISA)-based assay specific for HA. No HA was detected in strains that were members of the emergent clade, i.e., negative for the HA capsule locus *hasABC*, as expected (Fig. 5C). Strains outside the emergent clade all produced clearly detectable, albeit variable, levels of HA.

The HA capsule has been shown to be a critical virulence factor, particularly in the resistance of different *emm*-type GAS strains to phagocytosis (29–35). Surprisingly, given the differences in the capsule phenotypes of clade-associated and non-clade-associated strains, all strains were equally able to survive and multiply in whole human blood (Fig. 5D).

We hypothesized that the emergent clade-associated strains might demonstrate enhanced persistence, related to loss of capsule and increased exposure of streptococcal surface binding proteins. To test this, the ability of both clade-associated and non-clade-associated strains to adhere to and colonize plastic was measured. On uncoated plastic, the emergent clade-associated strains were significantly better able to adhere and colonize than non-clade-associated strains (Fig. 5E), confirming that the emergent clade had acquired a phenotype that may be advantageous to environmental transmission or persistence.

## DISCUSSION

The rise of *emm*89 iGAS in the United Kingdom coincided with the emergence and increased prevalence of a variant acapsular clade that differed from the rest of the *emm*89 population by six regions of core genome homologous recombination, providing the first direct evidence of multiple dynamic changes in the GAS core genome within a single *emm* type. Of these genomic regions, two were most notable: first, the absence of the *hasABC* locus, resulting in nonencapsulation, and, second, changes in the *nga*-*slo* locus with enhanced expression of these toxins. We hypothesize that the genome remodeling that occurred in the emergent clade variant provided a selective advantage that allowed it to outcompete other *emm*89 variants.

A high level of variation can occur within specific *emm*-type populations, but this is usually attributed to mobile genetic element-mediated DNA transfer such as bacteriophages or integrative conjugative elements (ICEs). The recombination events leading to the emergence of the new clade variant appeared to be through core genome homologous recombination, not associated with mobile genetic elements, the mechanism for which is not understood in naturally untransformable bacteria such as GAS (36). It is possible that the emergent clade-associated strains acquired heightened ability to recombine compared with other *emm*89 strains, potentially through the loss of capsule allowing enhanced exogenous DNA uptake or some other unknown mechanism arising from core genomic changes. The close proximity of SNPs within each region suggests that single recombination events occurred at each site, although the mechanism behind the recombination events is uncertain, and two or more sites may have recombined in a single event. A lower dN/dS ratio was observed within regions of recombination compared to the rest of the genome, indicating diverged donor lineages already selectively purged of deleterious mutations (17). The donor(s) may have been another GAS strain of a different *emm* type or possibly even another streptococcal species such as *S. dysgalactiae* subsp. *equisimilis*. Genetic exchange between GAS and *S. dysgalactiae* subsp. *equisimilis* has been previously described, although prophage or ICE mediated (37). Interestingly, although separated by ~53 to 129 kb from each other, the six regions are located within 239 to 261 kb of the origin of replication. The origin of replication as a hot spot for homologous recombination has been observed for other bacteria, possibly due to an increase in DNA copy number surrounding the origin during replication and exponential growth (36, 38).

The genome remodeling that led to emergence of the new clade had a substantial impact on two virulence factor loci with clear phenotypic consequences. Although the phenotypic changes observed appear significant, we cannot exclude other factors that may have aided the success of the new clade variant, including potential subtle effects from one or more of the other regions of recombination or SNPs located elsewhere in the genome. Outside the six regions of recombination, 27 SNPs were shared by all clade-associated strains compared to all non-clade-associated strains (see Table S2 in the supplemental material). Eighteen of 27 of these SNPs were nonsynonymous changes, and 14 were predicted to affect protein structure and/or function (as predicted by SIFT Blink [39]). We could not determine any obvious negative or positive impact that these SNPs may have on pathogenicity, based on predicted functions of the proteins, although we cannot exclude a role, and further work is required to determine any possible contribution to the emergence of the new clade. Notably, there was evidence for selection outside the six regions of recombination: three clade-associated SNPs were found in *parE* (two of which were nonsynonymous) and two clade-associated SNPs were found in *pstA* (one nonsynonymous). While these genes were not predicted to be areas of recombination by Gubbins analysis, and neither encodes known virulence factors, a role in pathogenesis remains possible. We predict that, together with the regions of recombination identified, there are likely to be additional changes contributing to the success of the emergent clade in the *emm*89 population.

The absence of the HA capsule locus in the emergent clade variant *emm*89 was unexpected and distinct from any dynamic change previously reported in GAS. The HA capsule was believed to be required by other serotypes for full pathogenesis. The ability of acapsular *emm*89 to survive and indeed outcompete related encapsulated strains, along with the recent recognition that *emm*4 and *emm*22 strains are also acapsular (28), suggests that encapsulation is not as essential for pathogenesis by all strains as previously thought and may even provide an advantage. Whether acapsular status represents an adaptation to altered environment or host response is unknown, but so far, *emm*89 is the only *emm* type to include both capsular and acapsular genotypes. We do not know if the MRCA of the *emm*89 population was genetically capsular or acapsular; hence, it is unclear as to whether the emergent clade variant became acapsular through recombination with *emm*4, *emm*22, or even *S. dysgalactiae* subsp. *equisimilis* or whether other non-clade-associated strains became capsular through recombination with other encapsulated *emm* types.

The Bayesian analyses and root-to-tip correlation converged to estimate that the acapsular genotype/phenotype of *emm*89 has been in the population since at least the early 1990s, but the rise to domination was a more recent event, which was associated with a sudden increase in the incidence of iGAS. Although surprising, we do not know if the length of time between emergence and increase to dominance of this variant over other *emm*89 variants is atypical. Transmission rates and spread of GAS clones within the host population are not well understood, and work such as this has been limited so far. Acquisition of additional SNPs and/or the *speC/spd1*-associated prophage ΦM89.1 may have also contributed to the sudden increase of the acapsular clade variant within the population. Unknown host factors influencing the bacterial population should also not be excluded. It will be interesting to continue to monitor the *emm*89 population and expand the study globally.

Prevalence of noninvasive GAS infection is not subject to rigorous national surveillance; thus, we cannot rule out a specific association with invasive disease, although this seems unlikely as genomic differences were found in both invasive and noninvasive strains. There was also no indication of enhanced severity associated with invasive disease caused by the emergent clade-associated isolates, as indicated by 7- and 30-day case fatality rates. We hypothesize that the genomic changes acquired by clade-associated strains may have favored mucosal or fomite adherence and transmission, affecting the quantity rather than severity of invasive disease. This is supported by the observation that clade-associated strains demonstrated an enhanced ability to adhere to plastic compared with non-clade-associated strains.

During throat carriage, GAS can undergo inactivating mutations in the capsule locus that prevent capsule synthesis, but loss of capsule promotes both adhesion to epithelium and internalization into host pharyngeal cells (40). Such strains, however, lack virulence and are poorly able to survive in whole human blood, remaining fixed in a colonization state unable to cause invasive disease. In contrast, acapsular *emm*89 clade-associated GAS strains have acquired potential for long-term colonization through complete loss of capsule but manifestly retain the ability to cause invasive disease. Thus, long-term-colonized individuals may carry and transmit the emergent clade-associated *emm*89 strains capable of causing invasive disease. The acapsular nature of the emergent clade-associated strains and increased expression of SLO and NADase will enhance internalization and intracellular bacterial survival in epithelial cells, theoretically providing protection from natural antimicrobial peptides and antibiotic treatment.

The acquisition of a 36-kb genomic region that includes the *nga*-*slo* locus, possibly from M12, with enhanced toxin expression is thought to be responsible for the emergence and subsequent rapid global spread of M1T1 (19, 25). This mirrors our observation of the emergence of acapsular clade-associated *emm*89 as a leading cause of disease that had acquired a similar *nga*-*slo* locus as part of a 12-kb region of recombination (region 2). This region appears variable across several different *emm* types, and it is unclear at this stage whether enhanced *nga*-*slo* toxin production is due to a single or several polymorphisms within the *nga*-*slo* locus and promoter or a combination of multiple factors present in the surrounding 12-kb region and/or the entire genome.

Within the emergent clade-associated strains, we also identified a phage-like element, ΦM89.1, associated with two other virulence factors, a superantigen encoded by *speC* and a DNase encoded by *spd1*, which may also contribute to disease pathogenesis. ΦM89.1 was not, however, a ubiquitous feature of the emergent clade variant. Some non-clade-associated *emm*89 strains also carried *speC* and *spd1*, although they were associated with other prophages that can be found in other *emm* types of GAS. The presence of other superantigen genes, *speA*, *speK*, *speL*, *speM*, and *ssa*, varied between all *emm*89 strains attributable to the varying distribution of other mobile GAS prophages.

Although this was a United Kingdom-based study, *emm*89 GAS is increasing in prevalence worldwide (2, 6, 9, 12). Whether clade shift is occurring globally is as yet unclear, although we have identified at least one clade-associated genotype strain from Geneva, Switzerland. Standard molecular typing methods failed to distinguish the emergent acapsular clade variant from the previous *emm*89 population, though PCR-based surveillance (as detailed in Fig. S6 in the supplemental material) will allow continued enhanced surveillance of this important clade shift. *emm*89 GAS strains are widely considered to be capable of causing both skin and throat infections (41), as members of the so-called *emm* pattern group “E,” a grouping system related to the chromosomal organization of *emm* subfamily genes. The differences observed in the current study occurred without any change in *emm* gene organization and may affect patterns of GAS persistence and carriage in humans or, potentially, in the environment. The findings underline the need to reassess the population risks posed by human infection or environmental contamination by nonencapsulated GAS strains and support further investigation of potential sources of transmission based on circulating strain types.

## MATERIALS AND METHODS

### Epidemiology.

Epidemiological data were extracted from all invasive infections reported to the Public Health England reference laboratory from the United Kingdom between 1994 and 2013 which were associated with invasive (sterile-site) isolates. Prior to 1999, *emm*/M89 was designated PT4245 (42), and these infections were also included. Seven- and 30-day case fatality rates were obtained where data were available; prior to 2003, data could not be obtained accurately and so were excluded from the analysis. Patient vital status was derived from the Demographic Batch Service.

### Bacterial strains.

Thirty invasive and noninvasive *emm*89 GAS isolates from Imperial College Healthcare NHS Trust, West London, were cultured and stored between 1995 and 2011 (see Table S1 in the supplemental material). One hundred one additional *emm*89 isolates were obtained from strains submitted to the national reference laboratory. These were randomly selected to represent a 10-year period (2004 to 2013) and locations throughout the United Kingdom plus one isolate from Switzerland. Nine to 11 isolates per year were selected, with approximately equal numbers of noninvasive and invasive strains. All GAS isolates were cultured on Columbia horse blood agar (Oxoid, Basingstoke, United Kingdom) or in Todd-Hewitt liquid broth (Oxoid) at 37°C with 5% CO_2_.

### Whole-genome sequencing and phylogenetic analysis.

Multiplex paired-end Illumina sequencing was performed on 34 isolates at Imperial College London using Illumina MiSeq, generating 150-bp reads. The Illumina MiSeq-generated short read sequences of strain H293 were used with standard sequencing to generate a completed genome. Two independent assemblies of the sequence reads were carried out using the SPAdes assembler (43) (with k-mer sizes of 21, 33, and 55) and ABySS (44) (with a k-mer size of 63). The resulting contig sequences were combined using Zorro (http://www.lge.ibi.unicamp.br/zorro) and scaffolded against the complete sequence of the M12 MGAS9429 genome (EMBL accession no. CP000259) using ABACAS (Wellcome Trust Sanger Institute). Gap closure was carried out using the Gap5 program from the Staden package (45), yielding an assembly consisting of 10 contigs in one scaffold. Contigs were then joined by PCR and standard Sanger sequencing across gaps. Automated annotation was performed on the completed genome using Prokka (Victorian Bioinformatics Consortium [vicbioinformatics.com]), and the resulting annotations were manually verified.

Using SMALT (Wellcome Trust Sanger Institute), reads were mapped to the completed *emm*89 H293 genome (EMBL accession no. ERP002615 and HG316453.2), and single nucleotide polymorphisms (SNPs) were identified. Concatenated SNPs identified in the core genome were used to generate a maximum likelihood tree using RAXML (46). *De novo* assembly sequences were constructed using Velvet and SPAdes. Regions of SNP clustering and potential recombination were identified using Gubbins (47).

Path-O-Gen (http://tree.bio.ed.ac.uk/software/pathogen/) was used to conduct the linear regression of maximum likelihood root-to-tip distances against the year of sampling. The Bayesian software package BEAST (v1.7.4) (48) was used to investigate the temporal dynamics of the *emm*89 population. To estimate the substitution rates and times for divergences of internal nodes on the tree, a general time-reversible (GTR) model with a gamma correction for among-site rate variation was used. To identify the most suitable models, we compared the strict, lognormal-relaxed, and exponential-relaxed molecular clock models and coalescent constant, exponential growth, expansion growth, and Bayesian skyline tree models. For each, three independent chains were run for 100 million generations, with sampling every 10 generations. On completion, each model was checked for convergence, both by checking that exponential sequence scheme (ESS) values were greater than 200 for key parameters and by checking that independent runs had converged on similar results. Models, including exponential and expansion population, which failed to converge so were discarded. Models were compared for their fit to the data using Bayes factors based on the harmonic mean estimator as calculated by the program Tracer v1.4 from the BEAST package. The constant tree model along with the strict molecular clock to accommodate for rate variation among lineages was preferred. A burn-in of 10 million states was removed from each of the three independent runs of this model before combining the results from those runs with the logcombiner program from the BEAST package. A maximum clade credibility (MCC) tree was created from the resulting combined trees using the treeAnnotator program, also from the BEAST package.

### NADase.

NADase activity was measured as previously described (25). Briefly, overnight bacterial culture supernatant was serially diluted 2-fold in phosphate-buffered saline (PBS) and incubated with 0.67 mmol/liter NAD^+^ (Sigma-Aldrich, Dorset, United Kingdom) for 1 h at 37°C. Reaction mixtures were developed with 2 N NaOH and incubated in the dark for 1 h before being visualized at 360 nm. Activity was measured as the highest dilution capable of hydrolyzing NAD^+^. Where required, bacterial supernatant was also concentrated 10-fold with trichloroacetic acid (TCA)-acetone precipitation and then subjected to Western blotting and probed for NADase with rabbit anti-NADase (Abcam, Cambridge, United Kingdom).

### SLO activity.

SLO activity was measured as previously described (25). Briefly, GAS strains were cultured to an *A*_600_ of 0.25, and filtered culture supernatant was incubated with 20 mmol/liter dithiothreitol (DTT) for 10 min at room temperature. Two percent sheep erythrocytes suspended in PBS was added and incubated at 37°C for 30 min before centrifugation at 3,000 × *g* for 5 min. Supernatants were then transferred to a 96-well plate, and *A*_541_ was read. As a control, 20 µg of water-soluble cholesterol (Sigma-Aldrich) was added as a specific inhibitor of SLO. SLO activity was reported as that specific to SLO (*A*_541_ of the sample minus *A*_541_ in the presence of cholesterol) expressed as a percentage of the positive control (sheep erythrocytes incubated with H_2_O).

### Capsule assay.

GAS strains were streaked onto Columbia horse blood agar from frozen glycerol stocks and incubated overnight at 37°C and 5% CO_2_. Colonies (5 to 10) were then taken off the plates and suspended in 300 µl sterile 10 mM Tris, pH 7.5. An aliquot of this suspension was then serially diluted and plated to obtain the number of CFU. Hyaluronic acid capsule was then detected and quantified as previously described (26).

### Growth in whole human blood.

Growth in whole human blood was performed as previously described (49). Briefly, approximately 50 CFU of GAS was inoculated into 300 µl of freshly extracted heparinized human blood and incubated at 37°C for 3 hours with rotation. Final CFU were measured by plating onto blood agar. The multiplication factor was calculated by dividing the final CFU by the initial inoculum.

### Bacterial adhesion assay.

Bacterial adhesion assays were performed as previously described (50, 51) with minor modifications. Bacterial strains were cultured overnight before being diluted 1 in 100 in fresh Todd-Hewitt medium (Oxoid), and 100 µl was applied to uncoated 96-well polystyrene tissue culture plates and incubated at 37°C for 24 h. Plates were then washed three times in PBS and stained with 100 µl Gram’s crystal violet for 30 min. After extensive washing, the stain was solubilized in 100% ethanol and *A*_595_ was measured.

### Nucleotide sequence accession numbers.

The sequences of the 34 isolates subjected to multiplex paired-end Illumina MiSeq sequencing were deposited in the short read archive (EMBL accession no. ERP005815). WGS data from the additional 97 *emm*89 isolates sequenced using Illumina HiSeq were also deposited in the short read archive (EMBL accession numbers ERR485686 to ERR485692, ERR485694 to ERR485700, ERR485702 to ERR485777, ERR485823 to ERR485825, ERR485827, ERR485871, and ERR485872). The completed *emm*89 H293 genome was deposited under EMBL accession no. ERP002615 (SRA) and HG316453.2.

## SUPPLEMENTAL MATERIAL

Figure S1  Identification of six regions of recombination in emergent clade-associated strains compared to non-clade-associated strains. Six regions of recombination (R1 to R6) were identified in all emergent clade-associated strains (green) that were absent in all non-clade associated strains (orange) in relation to an outgroup (black circle, *emm*2 strain MGAS10270) using Gubbins analysis for SNP clustering and recombination prediction (48). Regions of recombination identified in each strain are shown as vertical red lines (indicating recombination on internal nodes) or blue lines (indicating recombination on terminal branches), and genome coordinates are given on the top line. Various repeat regions within the clustered regularly interspaced short palindromic repeat (CRISPR) region are indicated by “C.” Download Figure S1, TIF file, 2.6 MB

Figure S2  Temporal calibration of the evolution of the *emm*89 isolates. (A) Maximum clade credibility tree generated following temporal Bayesian analyses on core SNPs in the genome excluding regions of recombination. The tMRCA of the whole population was estimated to be 2 May 1970. (B) Linear regression of the root-to-tip distances was carried out using Path-O-Gen v1.4 (http://tree.bio.ed.ac.uk/software/pathogen/) with a best-fit root from the maximum likelihood tree and the dates of isolation. The plot contains straight-line best fit of the root-to-tip divergence for each of the isolates, with a correlation coefficient of 0.6240 and a slope of 2.39 × 10^−6^. The tMRCA for the whole population was estimated to be 20 October 1970, consistent with the Bayesian analyses. Download Figure S2, TIF file, 2.6 MB

Figure S3  The difference in NADase activity between strains is due to differences in NADase protein expression. (A) Western blotting results for NADase in supernatants for seven strains representing non-clade-associated *emm*89 strains (labeled “*emm*89 strains”) and seven strains representing emergent clade-associated *emm*89 strains. (B) The expression of NADase corresponds to the activity of NGA/NADase. High levels of expression seen in strains H395, H543, HM11, and H636 are due to mutations in *covR/S* or the regulator of *covR/S*, *rocA*. Download Figure S3, TIF file, 2.3 MB

Figure S4  Phylogenetic tree of the *nga/slo* region in available GAS genomes (*n* = 19) plus *emm*89 (non-clade-associated) and emergent clade-associated *emm*89 strains. Variation in this region is apparent between M types. Consistent with a previous observation, modern M1 and M12 cluster together in a separate clade and *emm*89 emergent clade-associated strains are associated with this clade. *emm*89 (non-clade-associated) strains, however, cluster with M2 strain MGAS10270 in a different clade. Download Figure S4, TIF file, 2.8 MB

Figure S5  Comparison of region 6 sequences among *emm*89, emergent clade-associated *emm*89, and *S. dysgalactiae* subsp. *equisimilis*. Homology between strains is indicated in red. The 157-bp region present in emergent clade-associated *emm*89 strains in place of the *hasABC* locus (red box) is identical to that found in *S. dysgalactiae* subsp. *equisimilis*, and in a position homologous to the two genes downstream, *2273* and *recF* share ~93% identity to clade-associated *emm*89 genes *01677* and *recF*. The genes upstream of the 157-bp region, *2271* and *2272*, share some similarity to *01672* and *01673* of clade-associated *emm*89 strains (paler red shading). The *S. dysgalactiae subsp. equisimilis* strain AC-2713 complete genome sequence was used for comparison (EMBL accession no. HE858529.1). ACT (Wellcome Trust Sanger Institute) was used to compare the three genomes. Download Figure S5, TIF file, 0.9 MB

Figure S6  Schematic representation of PCR for clade assignment. (A) Confirmation of the presence or absence of the *hasABC* locus and the presence or absence of the 157-bp sequence that is present in the emergent clade-associated *emm*89 strains in place of the *hasABC* locus. The first primer pair (blue arrows) (forward, 5′-GTTGACAAGCTAGCTCCATAAAGTC; reverse, 5′-CGGTTGTTTCAGCGAGAAATCC) amplified across the *hasABC* locus from surrounding genes SPYH293_01673 and SPYH293_01677. Non-clade-associated *emm*89 strains generated a product of 4,670 bp, whereas clade-associated *emm*89 strains generated a product of only 632 bp. The second primer pair (green arrows) (forward, 5′-CCACATGACTATAAAGTTGCTG; reverse, 5′-CTGATAACGGATAGGTCTGTG) amplified a region within *hasA* of 106 bp, and a product was generated only in non-clade-associated *emm*89 strains. The third primer pair (red arrows) (forward, 5′-GCAATTGACTTGCTCCTATG; reverse, 5′-GACTATTCCAAAGTGAGACG), amplified within the 157-bp region (red box), is present only in clade-associated *emm*89 strains in place of the *hasABC* locus and generates a product of 127 bp in clade-associated *emm*89 strains. (B) To further characterize strains as non-clade-associated *emm*89 or clade-associated *emm*89, a second region of difference was tested. This region (region 2) contains four genes between *slo* and *metB* that share only ~73% DNA identity between non-clade-associated *emm*89 and clade-associated *emm*89 strains. Primers were designed to amplify between SPYH293_00193 and *metB* using a forward primer specific to the non-clade-associated *emm*89 type of SPYH293_00193 (Spy_00193_A, orange forward arrow) (5′-TCCGTCAGCTGTTAATTTAC) or the clade-associated *emm*89 type of SPYH293_00193 (Spy_00193_B, purple forward arrow) (5′-CAGATCCATCGTTAGTACAC) and a common reverse primer that binds in the conserved *metB* gene (orange reverse arrow) (5′-CAATAGCGTTAACTCCAATG). Non-clade-associated *emm*89 strains will generate a product of 657 bp with Spy_00193_A primer and *metB* primer but no product with Spy_00193_B primer and *metB* primer. Clade-associated *emm*89 strains will generate a product of 617 bp with Spy_00193_B primer and *metB* primer but no product with Spy_00193_A primer and *metB* primer. Download Figure S6, TIF file, 1.4 MB

Table S1  Clinical strains used in this study. Table S1, DOCX file, 0.1 MB

Table S2  Clade-associated SNPs outside the six regions of recombination (R1 to R6). Table S2, DOCX file, 0.1 MB
