# Supplementary figures and images for: Emergence of a New Highly Successful Acapsular Group A Streptococcus Clade of Genotype emm89 in the United Kingdom
Source: mBio. 2015 Jul 14;6(4):e00622-15. doi: 10.1128/mBio.00622-15 (PMC4502227; doi:10.1128/mBio.00622-15)

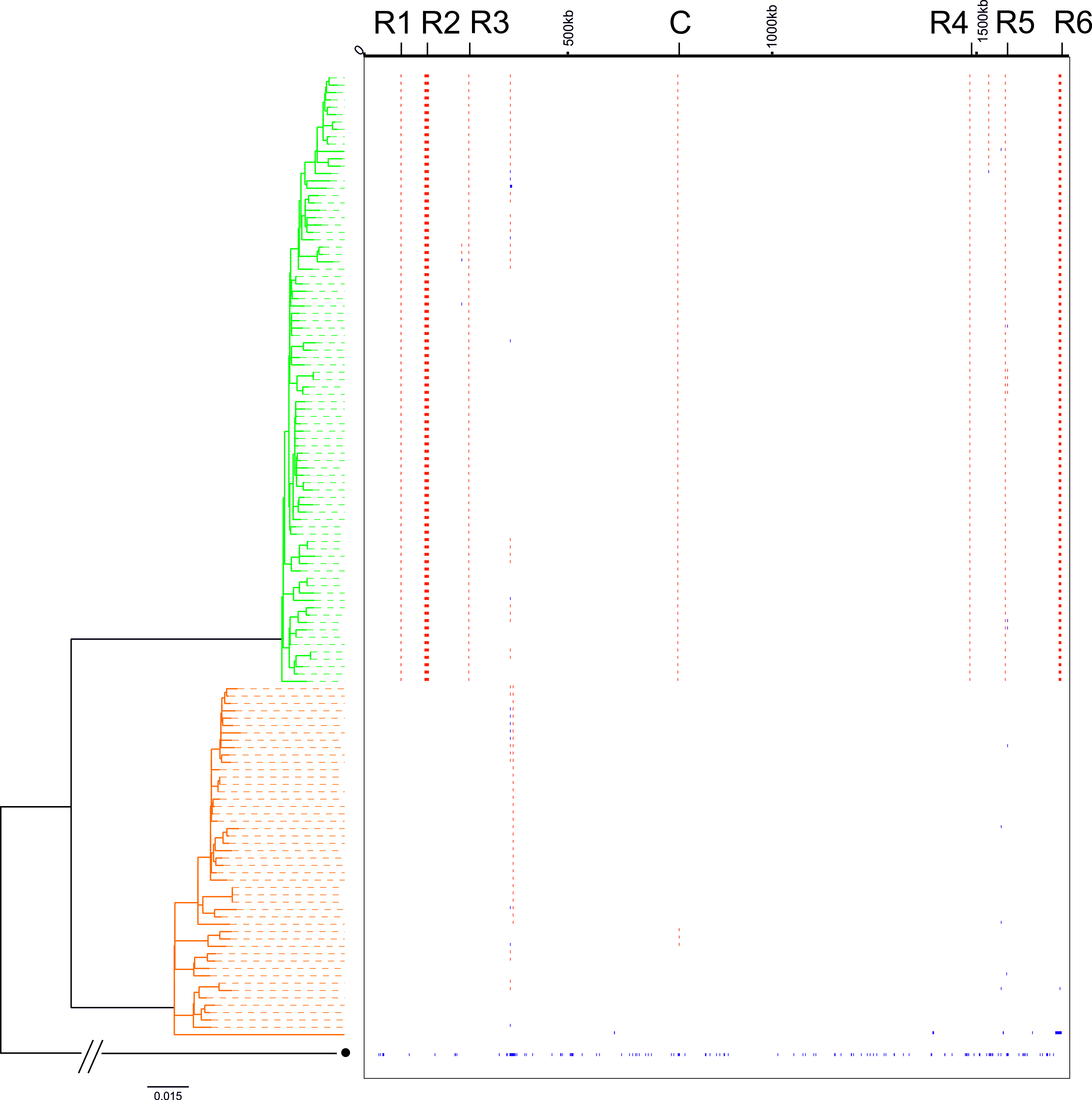

Supplement: Figure S1 — Identification of six regions of recombination in emergent clade-associated strains compared to non-clade-associated strains. Six regions of recombination (R1 to R6) were identified in all emergent clade-associated strains (green) that were absent in all non-clade associated strains (orange) in relation to an outgroup (black circle, emm2 strain MGAS10270) using Gubbins analysis for SNP clustering and recombination prediction (48). Regions of recombination identified in each strain are shown as vertical red lines (indicating recombination on internal nodes) or blue lines (indicating recombination on terminal branches), and genome coordinates are given on the top line. Various repeat regions within the clustered regularly interspaced short palindromic repeat (CRISPR) region are indicated by “C.” Download [file mbo004152392sf1.tif]

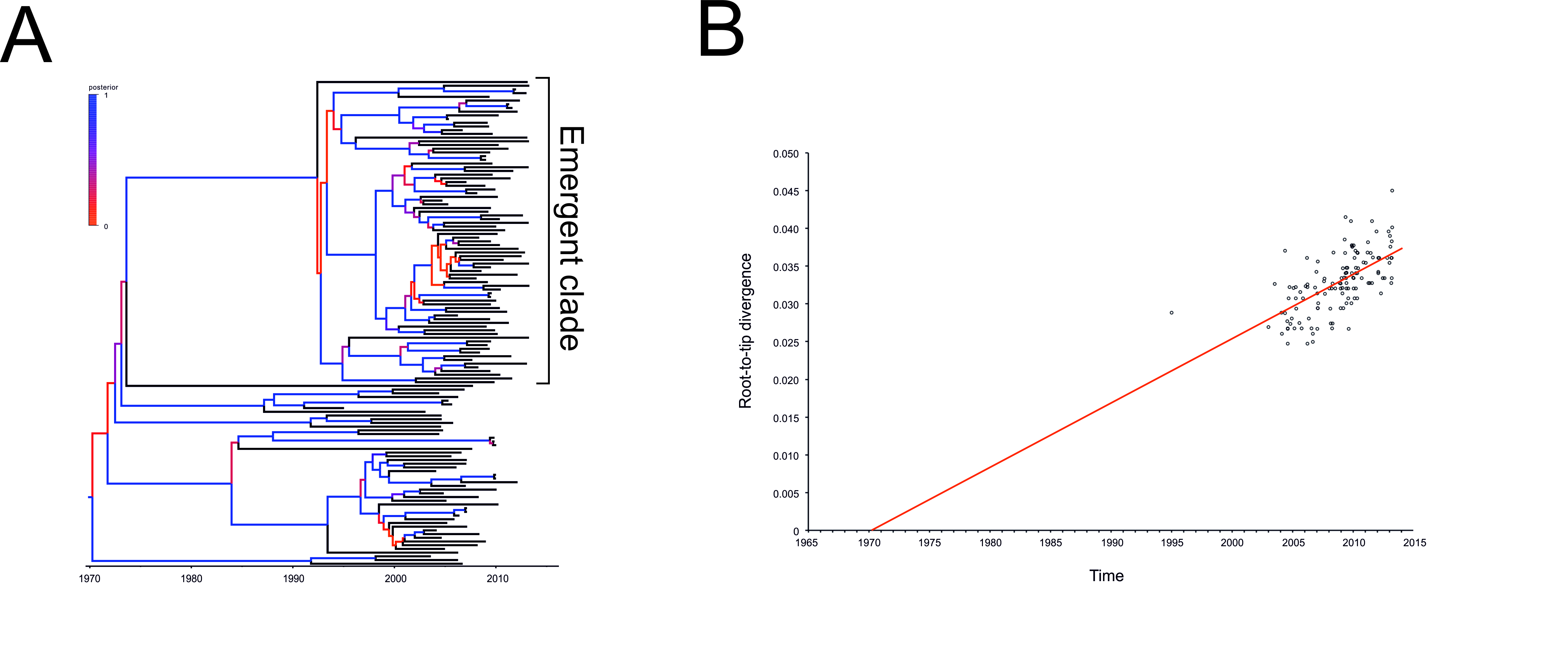

Supplement: Figure S2 — Temporal calibration of the evolution of the emm89 isolates. (A) Maximum clade credibility tree generated following temporal Bayesian analyses on core SNPs in the genome excluding regions of recombination. The tMRCA of the whole population was estimated to be 2 May 1970. (B) Linear regression of the root-to-tip distances was carried out using Path-O-Gen v1.4 (http://tree.bio.ed.ac.uk/software/pathogen/) with a best-fit root from the maximum likelihood tree and the dates of isolation. The plot contains straight-line best fit of the root-to-tip divergence for each of the isolates, with a correlation coefficient of 0.6240 and a slope of 2.39 × 10^−6^. The tMRCA for the whole population was estimated to be 20 October 1970, consistent with the Bayesian analyses. Download [file mbo004152392sf2.tif]

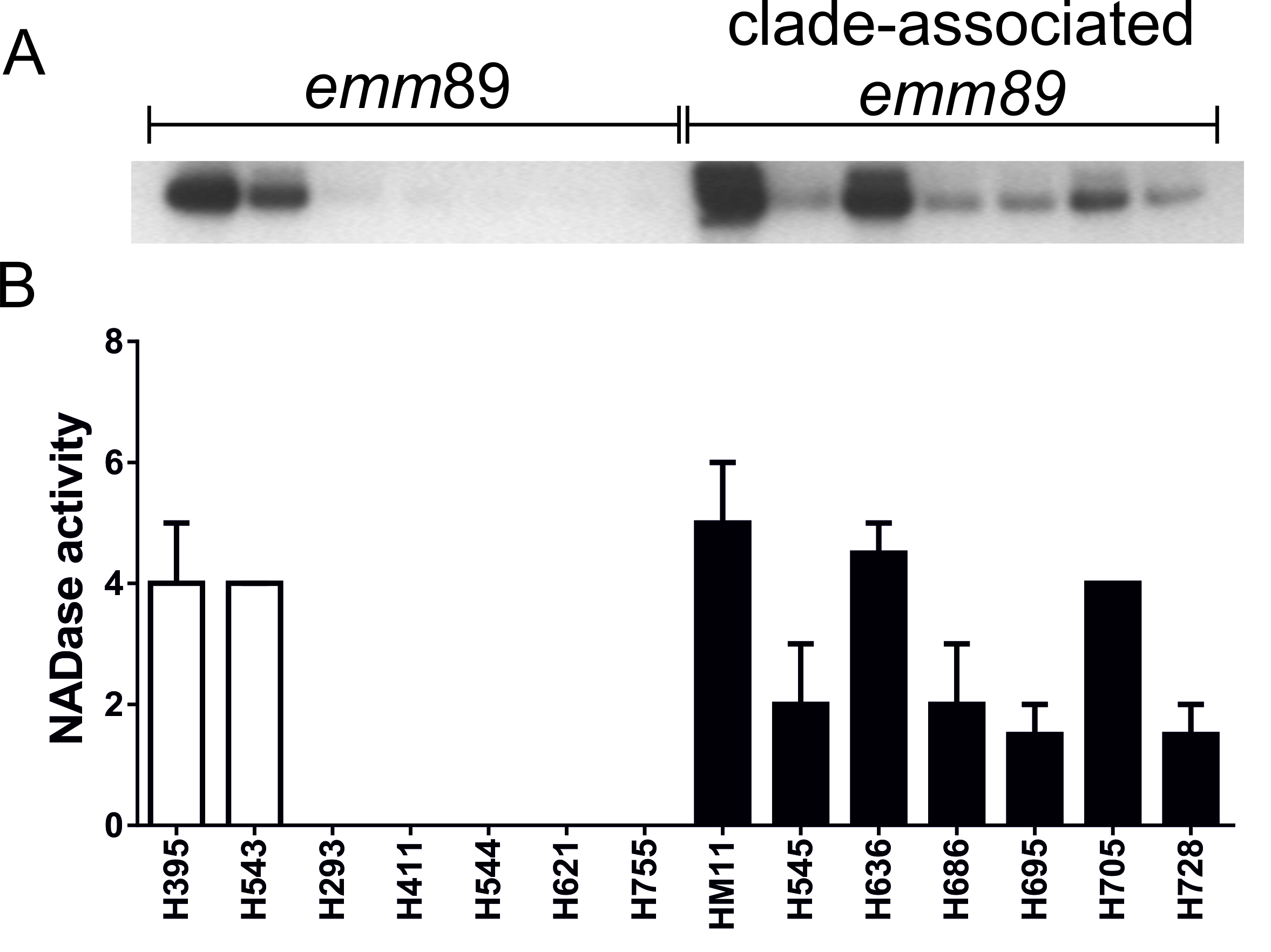

Supplement: Figure S3 — The difference in NADase activity between strains is due to differences in NADase protein expression. (A) Western blotting results for NADase in supernatants for seven strains representing non-clade-associated emm89 strains (labeled “emm89 strains”) and seven strains representing emergent clade-associated emm89 strains. (B) The expression of NADase corresponds to the activity of NGA/NADase. High levels of expression seen in strains H395, H543, HM11, and H636 are due to mutations in covR/S or the regulator of covR/S, rocA. Download [file mbo004152392sf3.tif]

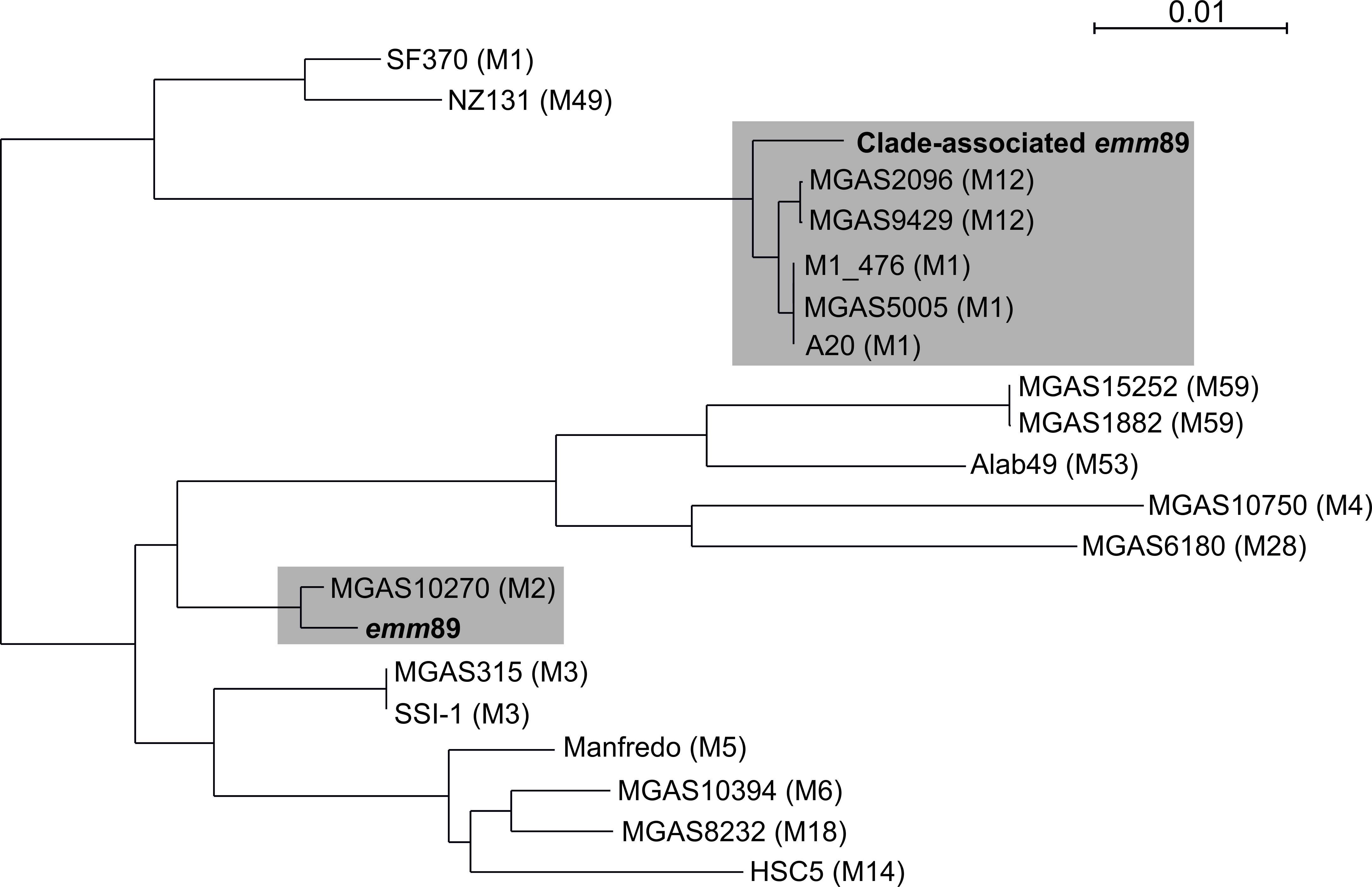

Supplement: Figure S4 — Phylogenetic tree of the nga/slo region in available GAS genomes (n = 19) plus emm89 (non-clade-associated) and emergent clade-associated emm89 strains. Variation in this region is apparent between M types. Consistent with a previous observation, modern M1 and M12 cluster together in a separate clade and emm89 emergent clade-associated strains are associated with this clade. emm89 (non-clade-associated) strains, however, cluster with M2 strain MGAS10270 in a different clade. Download [file mbo004152392sf4.tif]

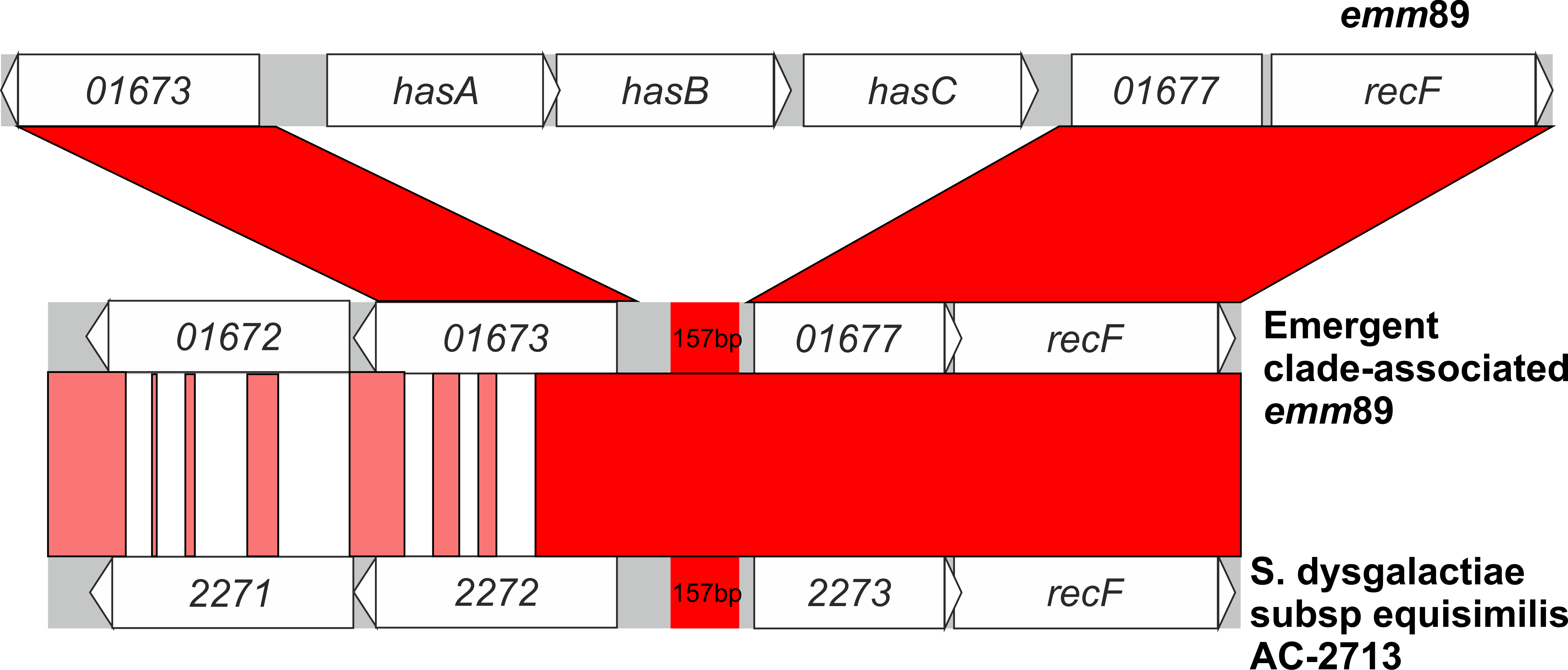

Supplement: Figure S5 — Comparison of region 6 sequences among emm89, emergent clade-associated emm89, and S. dysgalactiae subsp. equisimilis. Homology between strains is indicated in red. The 157-bp region present in emergent clade-associated emm89 strains in place of the hasABC locus (red box) is identical to that found in S. dysgalactiae subsp. equisimilis, and in a position homologous to the two genes downstream, 2273 and recF share ~93% identity to clade-associated emm89 genes 01677 and recF. The genes upstream of the 157-bp region, 2271 and 2272, share some similarity to 01672 and 01673 of clade-associated emm89 strains (paler red shading). The S. dysgalactiae subsp. equisimilis strain AC-2713 complete genome sequence was used for comparison (EMBL accession no. HE858529.1). ACT (Wellcome Trust Sanger Institute) was used to compare the three genomes. Download [file mbo004152392sf5.tif]

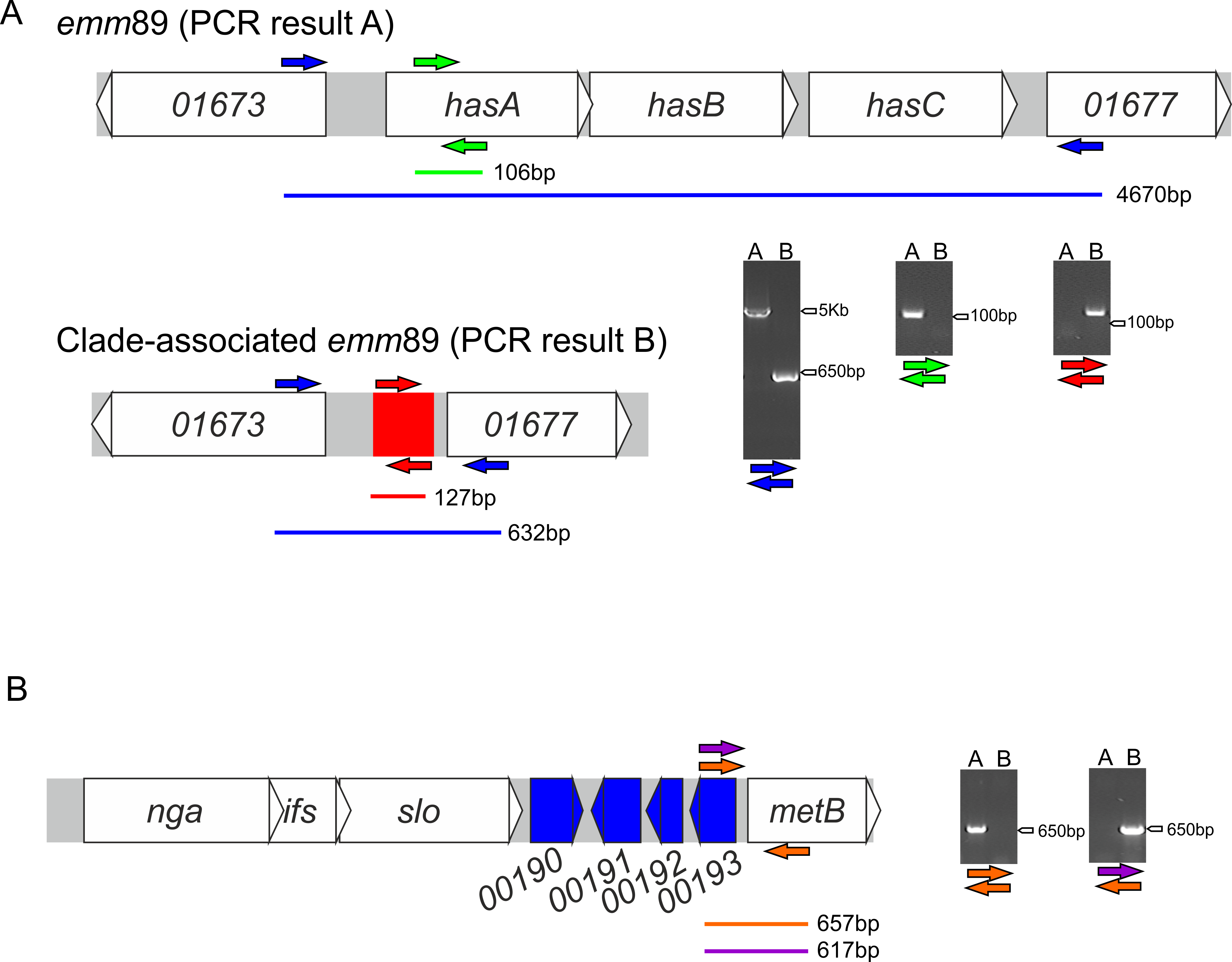

Supplement: Figure S6 — Schematic representation of PCR for clade assignment. (A) Confirmation of the presence or absence of the hasABC locus and the presence or absence of the 157-bp sequence that is present in the emergent clade-associated emm89 strains in place of the hasABC locus. The first primer pair (blue arrows) (forward, 5′-GTTGACAAGCTAGCTCCATAAAGTC; reverse, 5′-CGGTTGTTTCAGCGAGAAATCC) amplified across the hasABC locus from surrounding genes SPYH293_01673 and SPYH293_01677. Non-clade-associated emm89 strains generated a product of 4,670 bp, whereas clade-associated emm89 strains generated a product of only 632 bp. The second primer pair (green arrows) (forward, 5′-CCACATGACTATAAAGTTGCTG; reverse, 5′-CTGATAACGGATAGGTCTGTG) amplified a region within hasA of 106 bp, and a product was generated only in non-clade-associated emm89 strains. The third primer pair (red arrows) (forward, 5′-GCAATTGACTTGCTCCTATG; reverse, 5′-GACTATTCCAAAGTGAGACG), amplified within the 157-bp region (red box), is present only in clade-associated emm89 strains in place of the hasABC locus and generates a product of 127 bp in clade-associated emm89 strains. (B) To further characterize strains as non-clade-associated emm89 or clade-associated emm89, a second region of difference was tested. This region (region 2) contains four genes between slo and metB that share only ~73% DNA identity between non-clade-associated emm89 and clade-associated emm89 strains. Primers were designed to amplify between SPYH293_00193 and metB using a forward primer specific to the non-clade-associated emm89 type of SPYH293_00193 (Spy_00193_A, orange forward arrow) (5′-TCCGTCAGCTGTTAATTTAC) or the clade-associated emm89 type of SPYH293_00193 (Spy_00193_B, purple forward arrow) (5′-CAGATCCATCGTTAGTACAC) and a common reverse primer that binds in the conserved metB gene (orange reverse arrow) (5′-CAATAGCGTTAACTCCAATG). Non-clade-associated emm89 strains will generate a product of 657 bp with Spy_00193_A primer and metB primer but no produc [file mbo004152392sf6.tif]
