# Supplementary material for: Emergence of a New Highly Successful Acapsular Group A Streptococcus Clade of Genotype emm89 in the United Kingdom
Source: mBio. 2015 Jul 14;6(4):e00622-15. doi: 10.1128/mBio.00622-15 (PMC4502227; doi:10.1128/mBio.00622-15)
Supplement: Table S1 — Clinical strains used in this study. [file mbo004152392st1.docx]

**Supplementary Table S1.** Clinical strains used in this study.

| **Strain Name** | **Geographical place of isolation** | **Sample Date** | **Sample Type** | **Clade^a^** |
| --- | --- | --- | --- | --- |
| H293 | UK; W London | 1995 | Invasive | - |
| H395 | UK; W London | 2003 | Invasive | - |
| H411 | UK; Stockport | 2003 | Invasive | - |
| H1002 | UK; Sheffield | 2004 | Non-invasive | - |
| H1003 | UK; Exeter | 2004 | Non-invasive | - |
| H1004 | UK; London | 2004 | Non-invasive | - |
| H1005 | UK; Sunderland | 2004 | Invasive | - |
| H1006 | UK; Portsmouth | 2004 | Invasive | - |
| H1007 | UK; Gloucester | 2004 | Invasive | - |
| H1008 | UK; Torquay | 2004 | Non-invasive | - |
| H1009 | UK; Southport | 2004 | Non-invasive | - |
| H1011 | UK; Nottingham | 2004 | Invasive | - |
| H543 | UK; Surrey | 2004 | Invasive | - |
| H544 | UK; Belfast | 2004 | Invasive | - |
| H1012 | UK; Cornwall | 2005 | Invasive | - |
| H1013 | UK; Kings Lynn | 2005 | Invasive | - |
| H1014 | UK; Cardiff | 2005 | Invasive | + |
| H1015 | UK; Birmingham | 2005 | Invasive | - |
| H1016 | UK; London | 2005 | Non-invasive | + |
| H1017 | UK; Carmarthen | 2005 | Non-invasive | - |
| H1018 | UK; Birmingham | 2005 | Non-invasive | - |
| H1020 | UK; Tyne | 2005 | Non-invasive | - |
| H1021 | UK; Birmingham | 2005 | Non-invasive | - |
| H545 | Switzerland; Geneva | 2005 | Non-invasive | + |
| H1022 | UK; Tyne | 2006 | Non-invasive | - |
| H1023 | UK; Worcester | 2006 | Invasive | - |
| H1024 | UK; Bristol | 2006 | Non-invasive | - |
| H1025 | UK; Shrewsbury | 2006 | Non-invasive | + |
| H1026 | UK; Aylesbury | 2006 | Invasive | - |
| H1027 | UK; Salisbury | 2006 | Non-invasive | - |
| H1028 | UK; Southampton | 2006 | Invasive | - |
| H1029 | UK; Barnsley | 2006 | Invasive | - |
| H1030 | UK; Carshalton | 2006 | Non-invasive | + |
| H1031 | UK; Wirral | 2006 | Invasive | - |
| H1032 | UK; Tyne | 2007 | Invasive | - |
| H1033 | UK; Liverpool | 2007 | Invasive | - |
| H1034 | UK; Margate | 2007 | Non-invasive | + |
| H1035 | UK; Dundee | 2007 | Invasive | - |
| H1036 | UK; Camberley | 2007 | Non-invasive | - |
| H1037 | UK; Camberley | 2007 | Non-invasive | - |
| H1038 | UK; Bath | 2007 | Invasive | - |
| H1039 | UK; Barnstaple | 2007 | Invasive | - |
| H1040 | UK; Birmingham | 2007 | Non-invasive | + |
| H1041 | UK; Salisbury | 2007 | Non-invasive | - |
| H1042 | UK; Chichester | 2008 | Non-invasive | + |
| H1043 | UK; London | 2008 | Invasive | + |
| H1044 | UK; London | 2008 | Invasive | - |
| H1045 | UK; Portsmouth | 2008 | Invasive | + |
| H1046 | UK; Keighley | 2008 | Non-invasive | + |
| H1047 | UK; Newcastle | 2008 | Non-invasive | - |
| H1048 | UK; Hull | 2008 | Non-invasive | + |
| H1049 | UK; Brighton | 2008 | Invasive | + |
| H1050 | UK; Kent | 2008 | Invasive | + |
| H1051 | UK; Torquay | 2008 | Non-invasive | - |
| H621 | UK; W London | 2008 | Invasive | - |
| H1052 | UK; Chichester | 2009 | Invasive | + |
| H1053 | UK; Camberley | 2009 | Non-invasive | + |
| H1054 | UK; Essex | 2009 | Invasive | + |
| H1055 | UK; Crawley | 2009 | Non-invasive | + |
| H1056 | UK; Doha | 2009 | Non-invasive | + |
| H1057 | UK; North Yorkshire | 2009 | Invasive | + |
| H1058 | UK; Barnstaple | 2009 | Non-invasive | + |
| H1059 | UK; Swindon | 2009 | Invasive | + |
| H1060 | UK; Sutton-In-Ashfield | 2009 | Non-invasive | + |
| H1061 | UK; Sheffield | 2009 | Invasive | - |
| H636^b^ | UK; W London | 2009 | Invasive | + |
| H637 | UK; W London | 2009 | Invasive | + |
| H657 | UK; W London | 2009 | Invasive | + |
| H671 | UK; W London | 2009 | Non-Invasive | + |
| H686 | UK; W London | 2009 | Non-Invasive | + |
| H692 | UK; W London | 2009 | Non-Invasive | + |
| H695 | UK; W London | 2009 | Non-Invasive | + |
| H705 | UK; W London | 2009 | Non-Invasive | + |
| H711 | UK; W London | 2009 | Non-Invasive | + |
| H728 | UK; W London | 2009 | Invasive | + |
| H740^c^ | UK; W London | 2009 | Non-Invasive | - |
| H741 | UK; W London | 2009 | Non-Invasive | - |
| H744 | UK; W London | 2009 | Non-Invasive | + |
| HM11 | UK; W London | 2009 | Non-Invasive | + |
| HM42 | UK; W London | 2009 | Non-Invasive | + |
| HM59 | UK; W London | 2009 | Non-Invasive | + |
| HM61 | UK; W London | 2009 | Non-Invasive | + |
| HM66 | UK; W London | 2009 | Non-Invasive | + |
| HM98 | UK; W London | 2009 | Non-Invasive | + |
| HM100 | UK; W London | 2009 | Non-Invasive | + |
| H1062 | UK; Blackpool | 2010 | Invasive | - |
| H1063 | UK; West midlands | 2010 | Invasive | + |
| H1064 | UK; Crawley | 2010 | Non-invasive | + |
| H1065 | UK; Leeds | 2010 | Invasive | - |
| H1066 | UK; Bury St Edmunds | 2010 | Non-invasive | + |
| H1067 | UK; Bury St Edmunds | 2010 | Non-invasive | + |
| H1068 | UK; Tyne and Wear | 2010 | Non-invasive | + |
| H1069 | UK; Oldham | 2010 | Invasive | + |
| H1070 | UK; Glasgow | 2010 | Non-invasive | + |
| H1071 | UK; Brighton | 2010 | Invasive | + |
| H755 | UK; W London | 2010 | Non-Invasive | - |
| H771 | UK; W London | 2010 | Non-Invasive | + |
| H773 | UK; W London | 2010 | Non-Invasive | + |
| H774 | UK; W London | 2010 | Non-Invasive | + |
| H788 | UK; W London | 2010 | Non-Invasive | + |
| H803 | UK; W London | 2010 | Non-Invasive | + |
| H804 | UK; W London | 2010 | Non-Invasive | + |
| H1072 | UK; Crawley | 2011 | Non-invasive | + |
| H1073 | UK; Carlisle | 2011 | Non-invasive | + |
| H1074 | UK; Sunderland | 2011 | Non-invasive | + |
| H1075 | UK; Bristol | 2011 | Invasive | + |
| H1076 | UK; Peterborough | 2011 | Invasive | + |
| H1077 | UK; Tyne and Wear | 2011 | Invasive | + |
| H1078 | UK; Dorchester | 2011 | Invasive | + |
| H1079 | UK; Bristol | 2011 | Invasive | + |
| H1080 | UK; Bury St Edmunds | 2011 | Non-invasive | + |
| H1081 | UK; Tyne | 2011 | Non-invasive | + |
| H1082 | UK; Kilmarnock | 2012 | Invasive | - |
| H1083 | UK; Worcester | 2012 | Non-invasive | + |
| H1084 | UK; London | 2012 | Invasive | + |
| H1085 | UK; Blackpool | 2012 | Non-invasive | + |
| H1087 | UK; Essex | 2012 | Invasive | + |
| H1088 | UK; Gorleston | 2012 | Non-invasive | + |
| H1089 | UK; Coventry | 2012 | Invasive | + |
| H1090 | UK; Wishaw | 2012 | Invasive | + |
| H1091 | UK; Tyne | 2012 | Non-invasive | + |
| H1092 | UK; Chelmsford | 2013 | Invasive | + |
| H1093 | UK; Wirral | 2013 | Invasive | + |
| H1094 | UK; Crawley | 2013 | Non-invasive | + |
| H1095 | UK; Kent | 2013 | Invasive | + |
| H1096 | UK; Exeter | 2013 | Non-invasive | + |
| H1097 | UK; Plymouth | 2013 | Non-invasive | + |
| H1098 | UK; Taunton | 2013 | Non-invasive | + |
| H1099 | UK; Tyne | 2013 | Non-invasive | + |
| H1100 | UK; Cornwall | 2013 | Invasive | + |
| H1101 | UK; Edinburgh | 2013 | Invasive | + |

^a^Member of the emergent acapsular clade

^b^H636 and H637 were from the same patient isolated from two sites.

^c^H740 and H741 were separate colonies isolated from the same patient.
