# Supplementary material for: Emergence of a New Highly Successful Acapsular Group A Streptococcus Clade of Genotype emm89 in the United Kingdom
Source: mBio. 2015 Jul 14;6(4):e00622-15. doi: 10.1128/mBio.00622-15 (PMC4502227; doi:10.1128/mBio.00622-15)
Supplement: Table S2 — Clade-associated SNPs outside the six regions of recombination (R1 to R6). [file mbo004152392st2.docx]

**Supplementary Table S2.** Clade-associated SNPs outside the six regions of recombination (R1-R6).

| **Position in H293** | **Gene locus** | **Gene** | **Product** | **S/NS** | **Ref** | **SNP** | **Ref aa** | **SNP aa** | **Codon** | **Tolerated?^1^** |
| --- | --- | --- | --- | --- | --- | --- | --- | --- | --- | --- |
| 54909 | SPYH293_00069 | ruvB | Holliday junction ATP-dependent DNA helicase | S | T | C |  |  |  |  |
| 180849 | SPYH293_00208 | perR | Peroxide-responsive repressor perR | N | C | T | Proline | Serine |  | **No** |
| 286517 | SPYH293_00313 | atmE | Methionine ABC transporter permease protein | S | C | T |  |  |  |  |
| 311370 | SPYH293_00334 |  | OxaA-like protein precursor | S | G | T |  |  |  |  |
| 430162 | SPYH293_00465 | drrA | Daunorubicin/doxorubicin resistance ATP-binding | N | G | T | Glutamic acid | Aspartic acid | 142/300 | Yes |
| 538304 | SPYH293_00568 | agaS | galactosamine-6-phosphate isomerase | N | G | A | Cysteine | Tyrosine | 396/399 | **No** |
| 573597 | Intergenic | - | - | - | T | A |  |  |  |  |
| 671683 | SPYH293_00692 |  | 5'-nucleotidase (pseudogene in all strains) | - | G | T |  |  |  |  |
| 704888 | SPYH293_00725 | parE | DNA topoisomerase 4 subunit B | N | A | C | Lysine | Asparagine | 4/649 | **No** |
| 705401 | SPYH293_00725 | parE | DNA topoisomerase 4 subunit B | S | C | A |  |  |  |  |
| 706707 | SPYH293_00725 | parE | DNA topoisomerase 4 subunit B | N | G | A | Aspartic acid | Asparagine | 611/649 | **No** |
| 802297 | SPYH293_00817 | srtB | serine/threoninedehydratase | N | C | T | Proline | Serine | 632/901 | **No** |
| 823711 | SPYH293_00844 | aphA | Class B acid phosphatase precursor | N | G | A | Alanine | Threonine | 206/243 | **No** |
| 830086 | SPYH293_00851 |  | hypothetical protein | N | C | A | Alanine | Serine | 72/155 | Yes |
| 937839 | SPYH293_00956 | pstA | Phosphate transport system permease protein pstA | S | C | T |  |  |  |  |
| 937975 | SPYH293_00956 | pstA | Phosphate transport system permease protein pstA | N | G | A | Threonine | Isoleucine | 16/295 | **No** |
| 980305 | SPYH293_00996 | glgP | Maltodextrin phosphorylase | N | A | G | Isoleucine | Threonine | 85/754 | **No** |
| 1065342 | SPYH293_01073 | prsA.1 | Foldase protein prsA precursor | S | G | T |  |  |  |  |
| 1070691 | SPYH293_01077 |  | Competence protein | N | C | T | Glycine | Serine | 163/200 | **No** |
| 1084091 | SPYH293_01091 |  | potassium transport protein Kup | N | C | T | Glutamic acid | Lysine | 525/666 | **No** |
| 1114485 | SPYH293_01117 | phrA | Deoxyribodipyrimidine photo-lyase | N | C | T | Aspartic acid | Asparagine | 126/469 | **No** |
| 1225989 | SPYH293_01223 |  | Serine/threonine-protein kinase | S | C | T |  |  |  |  |
| 1236683 | SPYH293_01232 | atoB.2 | Acetyl-CoA acetyltransferase | N | A | G | Glutamic acid | Glycine | 191/395 | Yes |
| 1263870 | SPYH293_01258 |  | hypothetical protein | N | C | A | Valine | Phenyalanine | 55/71 | **No** |
| 1342652 | SPYH293_01340 |  | hypothetical protein | N | C | T | Alanine | Threonine | 33/79 | **No** |
| 1408093 | SPYH293_01398 | recD | Exodeoxyribonuclease V alpha chain | N | G | A | Methionine | Isoleucine | 199/817 | Yes |
| 1723807 | SPYH293_01690 | rmlH | Ribosomal RNA large subunit methyltransferase H | N | C | A | Glutamic acid | Aspartic acid | 37/159 | **No** |

^1^Tolerated; predicted tolerance of variant amino acid (aa) by SIFT (40)
